# Rise of prediagnostic molecular profiling in inflammatory bowel disease—can we close the door before the horse has bolted?

**DOI:** 10.1136/gutjnl-2025-336049

**Published:** 2026-02-13

**Authors:** Nick Powell

**Affiliations:** 1 Division of Digestive Diseases, Imperial College London, London, UK

**Keywords:** IBD BASIC RESEARCH, IBD CLINICAL, IBD

## Abstract

Inflammatory bowel disease (IBD) is typically diagnosed after the onset of symptoms in the context of established, characteristic patterns of intestinal inflammation. However, there is now substantial evidence pointing to a prolonged, biologically active preclinical phase of disease. Analysis of archived biological samples from large-scale longitudinal cohort studies of healthy individuals, some of whom develop incident IBD, has identified different molecular features that can be detected many years before clinical presentation. These include increased titres of antimicrobial and autoreactive antibodies and perturbations in a complex network of circulating, immunologically active proteins. As well as affording ‘diagnostic’ opportunities to identify individuals destined to develop IBD, an integrated view of these multiple different molecular features enables speculation of potential proximal drivers of preclinical IBD. Consistently recognised associations include dysregulated mononuclear phagocyte–lymphocyte interactions, augmented chemotaxis, frequently relating to interferon-γ–driven chemokine programmes and evidence of early tissue injury, such as increased circulating extracellular matrix components and metalloproteinases. Increased levels of circulating antibacterial and antiviral antibody responses hint towards disordered host–microbe interactions as potential prime triggers for the transition between health and early disease, although it is possible that these serological responses are an epiphenomenon linked to early mucosal damage and microbial translocation. There is now a timely opportunity to develop these different molecular features into scalable and clinically tractable biomarker panels to detect preclinical disease and enable strategies to proactively intercept IBD before it even develops.

 Preclinical detection of diseases, including inflammatory bowel disease (IBD), is a hot topic in medicine. Recent studies, and some dating back over 20 years, have identified molecular features that are enriched years in advance of symptom onset or a formal diagnosis of IBD (table 1). These predictive studies have been enabled through access to biological samples from large cohort studies of healthy individuals who have been followed up over long time periods until incident cases of IBD have emerged. For example, the Proteomic Evaluation and Discovery in an IBD Cohort of Tri-service Subjects cohort identified incident cases of IBD from the Department of Defence Medical Surveillance System, which holds data for over 7 million members who have served in the US armed forces and includes a repository of >50 million longitudinally acquired serum samples, which were initially collected to test for HIV.^1^ In addition, specifically designed prospective studies are now reporting, such as the groundbreaking Crohn’s and Colitis Genetic, Environmental, Microbial) project, which recruited over 5000 healthy siblings of Crohn’s disease (CD) patients and has prospectively defined over 100 incident cases of CD.^2^

**Table 1:** Major studies identifying molecular features associated with preclinical IBD

Author	Cohort	Molecular feature	Platform	Findings in preclinical IBD
Israeli *et al* ^3^	IDF Medical Corps Serum Repository	Serum ASCA and pANCA	Targeted ELISA and IIF	Increased serum antibodies; ASCA in CD and increased pANCA in UC up to 77 months prior to diagnosis.
Schaik *et al* ^4^	EPIC	Anti-microbial antibodies	Prometheus ELISA test	Combinations of ASCA, pANCA, anti-CBir and anti-OmpC predicted development of UC and CD
Choung *et al *^5^	PREDICTS cohort	Anti-microbial antibodies	Prometheus ELISA test	Anti-microbial antibodies (anti-ASCA, anti-OmpC, anti-CBir, anti-Fla2 and anti-FlaX) predate CD diagnosis.
Lee *et al* ^31^	CCC-GEM	Anti-microbial antibodies	Prometheus ELISA test	Increased ASCA, anti-A4-Fla2, anti-FlaX, anti-CBir and anti-OmpC in individuals who went on to develop CD.
Bourgonje *et al* ^37^	Lifelines Cohort Study	Blood antibody repertoire	PhIP-seq	Individuals destined to subsequently develop CD exhibited increased antibody reactivity to exotoxins from *Staphylococcus aureus*. Individuals going on to develop UC had increased responses against cell wall and secreted proteins of *S. aureus* and *Escherichia coli*.
Nandy *et al* ^7^	PREDICTS CCC-GEM	Antiviral serology	VirScan Anti-EBV nuclear antigen ELISA	Increased seropositivity to EBV in preclinical phase of CD up to 5–7 years prior to diagnosis.
Livanos *et al* ^21^	PREDICTS cohort	Anti-integrin αvβ6 antibodies	Targeted ELISA	Anti-integrin αvβ6 antibodies present in serum up to 10 years prior to diagnosis of UC
Mortha *et al* ^22^	PREDICTS cohort	Anti-GM-CSF antibodies	Targeted ELISA	Anti-GM-CSF antibodies detected in serum up to 6 years prior to diagnosis of CD, and were associated with a complicated disease course
Gaifem *et al* ^30^	PREDICTS cohort	Glycosylation signature of circulating antibodies	UPLC	Lower glycosylation of IgG2 Fc domains detected in serum of individuals who subsequently developed CD.
Torres *et al* ^20^	PREDICTS cohort	Circulating anti-microbial antibodies and protein	Prometheus anti-microbial antibody panel SomaLogic Multiplex proteome profiling	A statistical model incorporating anti-microbial antibodies and a panel of circulating proteins was predictive of development of either CD or UC. Increased abundance of anti-flagellin X and ASCA-IgA in preclinical CD up to 5 years pre-diagnosis. Increased abundance of pANCA in UC. Serum proteins associated with preclinical CD across time points measured included CRP, C5, Trypsin-2, serum Amyloid P component, osteomodulin and aggrecan. Proteins associating with preclinical UC included IL-11RA and LAG3
Granno *et al* ^8^	MDC and NSHDS	Protein signature	Olink Proteomics proximity extension assay	Nine proteins in discovery and validation cohort were associated with preclinical CD (increased CXCL9, IL6, MMP-10, CCL20, MDK and CXCL17, and reduced DNER, GPNMB and CX3CL1) 12 proteins in discovery and validation cohort were associated with preclinical UC (increased STAMBP, SIRT2, SCAMP3, ADAM8, GZMB, ABL1, CCL11, CD5, TNFRSF6B, MMP-10, CDKN1A and CXCL9).
Leibovitz *et al* ^9^	CCC-GEM	Protein signature	Olink Proteomics proximity extension assay	25 proteins associated with preclinical CD, comprising 15 upregulated proteins; CXCL9 (most discriminatory), HGF, PGLYRP1, OSM, CDCP1, MMP12, CLEC4D, MMP10, CPA1, CSF1, CLEC6A, CXCL11, MMP9, TREM1, MMP1 and 10 downregulated proteins; FAP, MEPE, DPP4, ZBTB16, CNTN1, HO-1, IL-17D, CD93, RAGE and IL-6RA.
Bergemalm *et al*^10^	Northern Sweden Health and Disease Study register cohort	Protein signature	Olink Proteomics proximity extension assay	Six proteins were increased in preclinical UC; MCP-1, CXCL9, SLAMF1, CCL11, MMP10 and CXCL11
Lochhead *et al*^38^	NHS and NHS II	Plasma IL-6 and hsCRP	Targeted ELISA and Immunoturbidimetry	Increased plasma concentrations of IL-6 and hsCRP in preclinical UC and CD
Hua *et al*^39^	NHS and NHS II PREDICTS	Metabolomic profile	LC-MS/HILIC	No replication of individual serum metabolites associated with preclinical CD or UC. At pathway level, preclinical CD was associated with biosynthesis of primary bile acids and steroid hormones, and metabolism of amino acids and nitrogen. Preclinical UC was associated with fatty acid biosynthesis.
Garay *et al*^40^	CCC-GEM	Microbiome composition	Sequencing of V4 hypervariable region of bacterial 16S rRNA	Microbiome risk score (MRS) in faecal microbiome was generated in individuals destined to develop CD. Most discriminatory taxa in the MRS were *Ruminococcus torques, Blautia, Colidextribacter, Oscillospiraceae* and *Roseburia*.

ASCA, anti-Saccharomyces cerevisiae antibodies; CCC-GEM, Crohn’s and Colitis Genetic, Environmental, Microbial; CD, Crohn’s disease; EBV, Epstein-Barr virus; ELISA, Enzyme linked Immunosorbent assay; GM-CSF, granulocyte macrophage colony-stimulating factor; HILIC, Hydrophilic Interaction Liquid Chromatography; IBD, inflammatory bowel disease; IL, Interleukin; LC-MS, Liquid chromatography mass spectrometry; MEPE, matrix extracellular phosphoglycoprotein; MMP, Metalloproteinase; OmpC, outer membrane protein C; OSM, Oncostatin M; PEDICTS, Proteomic Evaluation and Discovery in an IBD Cohort of Tri-service Subjects; PhIP-seq, Phage ImmunoPrecipitation Sequencing; TNFRSF, Tumour necrosis factor receptor superfamily; UC, Ulcerative colitis; UPLC, Ultra-Performance Liquid Chromatography.

Serological responses directed against bacteria, fungi and viruses are among the earliest and most consistently recognised features at the heart of many of these predictive studies. Over 20 years ago, an increased frequency of anti-*Saccharomyces cerevisiae* antibodies (ASCA) was detected in individuals many years in advance of developing CD.^3^ Subsequent studies have identified an increased frequency of antibodies reacting against bacterial flagellins (eg, anti-A4-Fla2, anti-FlaX and anti-CBir), the outer membrane protein C of *Escherichia coli* and exotoxins from *Staphylococcus aureus*.^2 4–6^ Antiviral antibody responses are also perturbed in the prediagnosis phase of CD, including an increased frequency of antibodies reacting to the EBNA-1 protein of Epstein-Barr Virus.^7^

It is possible that these preclinical antibody reactivities reflect aberrant host handling of commensals or pathogens, which then trigger dysregulation of finely tuned balances between immune activation and immune tolerance in the gut. An alternative view is that increased antimicrobial serological responses simply reflect persistent or exaggerated host exposure to intestinal commensals/pathogens, resulting from asymptomatic early mucosal lesions and impaired barrier function, which in turn leads to translocation of these organisms and immune activation. In the GEM study, antibacterial antibodies were a strong risk factor for developing CD independently of intestinal permeability,^2^ implying that these antibodies predate barrier defects. In keeping with microbes and their handling as proximal drivers of IBD, the GEM investigators observed shifts in the community composition of the intestinal microbiota prior to CD development.

A bird’s-eye view of the different molecules associated with the preclinical phase of IBD across different studies reveals some consistent patterns and enables speculation regarding potential immunological processes that might underpin disease initiation. These include prominence of chemotaxis, dysregulated immune interactions—mostly centred on lymphocytes and mononuclear phagocytes (MPs) and disruption of the extracellular matrix (ECM) (figure 1 and table 1). Preclinical formation of autoantibodies is also now established in a subgroup of patients with IBD.

**Figure 1:**
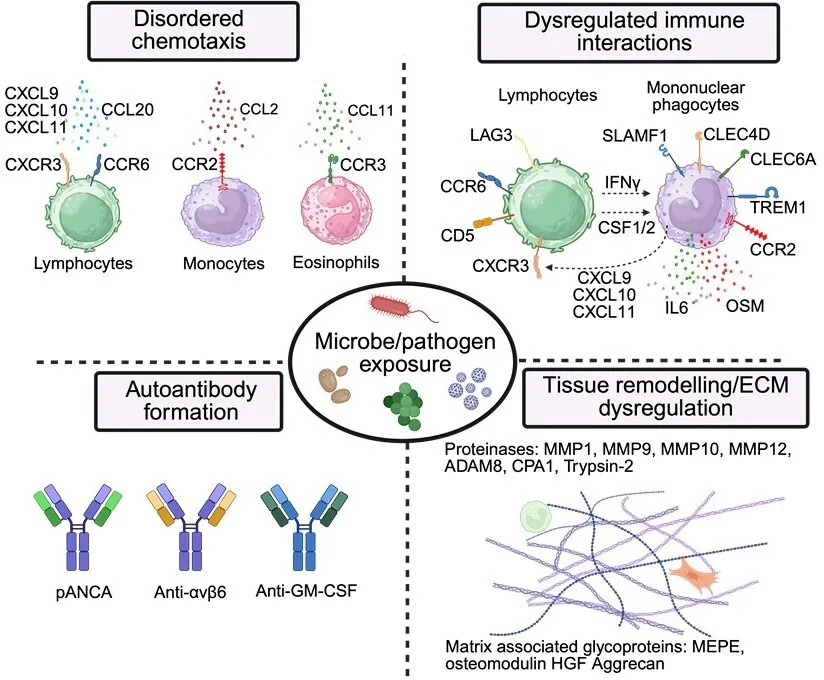
Potential mechanisms and processes implicated in the immunopathogenesis of IBD initiation based on molecular features that have been associated with preclinical IBD. ECM, extracellular matrix; IBD, inflammatory bowel disease inflammatory bowel disease.

Several studies have assessed peripheral blood proteomics in individuals who subsequently develop IBD, and one of the most consistently recognised immune features is upregulation of chemokines, which are an extended family of chemotactic cytokines with shared structures and overlapping functions. Together with integrins, chemokines regulate immune cell recruitment to selected tissues and sites of inflammation. Expression of the CXC family of chemokines CXCL9, CXCL10 and CXCL11 is consistently elevated in preclinical IBD.^8–10^ These closely related chemokines share functions, and all ligate the same receptor, CXCR3, expressed by effector lymphocytes, such as CD4^+^ Th1 T-cells and innate lymphoid cells (ILCs).^11^ Induction of all three of these chemokines is typically triggered by microbe/pathogen exposure and is a hallmark of the interferon-γ (IFNγ) response. Of the many thousands of IFNγ-induced genes, CXCL9, CXCL10 and CXCL11 are among the most strongly induced. Other chemokines that are expanded in preclinical IBD include CCL20, CCL11 and CXCL18. CCL20 is produced predominantly by epithelial cells and attracts lymphocytes after ligating its specific receptor CCR6, expressed by CD4^+^ Th17 and group 3 ILCs.^12^ Intriguingly, T-cells co-expressing CXCR3 and CCR6 adopt a pathogenic phenotype and are reactive to commensal bacteria.^13^ The eosinophil-active chemokine CCL11 (eotaxin-1) appears to be specifically enriched in the preclinical phase of ulcerative colitis (UC).^8 10^ Intriguingly, a third of patients with newly diagnosed UC have eosinophilia.^14^ CCL2 ligates CCR2 and plays a key role in the recruitment of monocytes and other MPs to inflammatory environments, often in response to microbial cues.^15^ Less is known about CXCL17, the most recently discovered chemokine. It may be a weak chemoattractant for some MPs and may potentiate the activity of other chemokines.^16 17^

It can be inferred from proteomic data that numerous immune interactions occur in preclinical IBD, and dialogue between MPs and lymphocytes features prominently. MPs, including monocytes, macrophages and dendritic cells, are sentinels of the immune system, sensing and responding to microbes to set the inflammatory tone of the local tissue environment. Together, they rapidly instruct adaptive immune responses and instigate proinflammatory responses. In keeping with an altered microbial encounter being a central proximal trigger for incident IBD, a number of pattern recognition receptors (PRRs) responsible for detecting microbes are increased in preclinical IBD, including TREM1 and the C type lectin domain proteins CLEC4D and CLEC6A (dectin-2) expressed by MPs.^9 10^ Although not a PRR, SLAMF1, which is expressed by MP, is overexpressed in preclinical UC, where it is required for Toll-like receptor 4 (TLR4) mediated responses against gram-negative bacteria, including induction of cytokine production.^18^ As well as being prominent producers of the chemokines CXCL9, CXCL10 and CXCL11, MPs are the chief producers of other cytokines that are elevated in preclinical IBD, including IL6 and oncostatin M. MPs are the target cells of other cytokines that are overexpressed in preclinical IBD, including CSF-1 (M-CSF), which promotes the differentiation and survival of MPs and triggers further production of CCL2, driving further local recruitment of monocytes.^19^ The production of CXCL9, CXCL10 and CXCL11 by MPs is likely driven by IFNγ produced locally by T-cells.

Increased expression of circulating proteases and metalloproteinases (MMPs) points to a prominent role for tissue remodelling and dysregulated interactions with the ECM in preclinical IBD and implies an antecedent inflammatory process. MMP1, MMP9, MMP10 and MMP12 are consistently increased in peripheral blood in both CD and UC across multiple studies.^8–10^ These enzymes can degrade tissue scaffolding proteins, including collagens, fibronectin, laminin and proteoglycans. Other proteases, such as ADAM8, CPA1 and trypsin-2, are also increased. Although stromal cells are key producers of MMPs and proteases, they can also be produced by MPs. Other glycoproteins with key functions regulating the ECM, such as matrix extracellular phosphoglycoprotein, osteomodulin, HGF and aggrecan, are also increased in preclinical IBD.^20^

Autoreactive antibody responses, against perinuclear antineutrophil cytoplasm (pANCA),^3^ the epithelial integrin αvβ6^21^ and granulocyte macrophage colony-stimulating factor (GM-CSF),^22^ are associated with preclinical IBD. It is not clear whether pANCA directly participates in disease pathogenesis or whether they are an immune response epiphenomenon. Notably, pANCA may stimulate neutrophils by ligating a cell surface membrane bound form of proteinase 3, which appears in primed neutrophils, which triggers neutrophil degranulation and reactive oxygen species generation, consistent with a directly pathogenic role.^23–25^ It has been speculated that pANCA may occur from molecular mimicry, reacting with host antigens after initially being elicited by infectious agents or luminal bacteria.^26^ Expansion of anti-αvβ6 antibodies predates diagnosis by many years in a substantial proportion of individuals who develop UC. αvβ6 is involved in ECM regulation, but its hallmark function is converting transforming growth factor β (TGFβ) into its active form.^27^ TGFβ is a crucial immunoregulatory cytokine that restrains intestinal inflammation.^28^ The paradigm of autoreactive antibodies neutralising host immunoregulatory proteins has gained further traction following identification of autoantibodies that neutralise the fundamentally important immunoregulatory cytokine IL10, in subset of patients with early onset CD.^29^ Anti-GM-CSF autoantibodies are present at least 6 years prior to diagnosis of CD.^22^ Disease-associated neutralising autoantibodies are directed against glycosylated post-translational modifications of the GM-SCF molecule. Patients harbouring anti-GM-CSF antibodies had distinct profiles of circulating inflammatory proteins, suggesting that GM-CSF neutralisation had specific impacts on host immune responses. The authors speculated that disruption of GM-CSF signalling impaired circuits between MPs and IFN-γ producing innate lymphocytes. Defects in glycosylation patterns of predisease associated antibodies have also been reported, including reduced Fc domain galactosylation of ASCA, which alters FcγR-dependent interactions between MPs and NK cells, driving proinflammatory NF-κB signalling and inflammasome activation.^30^

## Future perspectives

There is now a timely opportunity to prospectively define the performance characteristics of these different biomarkers and to develop scalable assays to accurately predict future IBD development. Antibody testing, proteomics, genomics and microbiome profiling are all potentially tractable approaches and multicomponent platforms, integrating the most discriminatory features together with clinical features, such as faecal calprotectin, C-reactive protein (CRP) and intestinal permeability tests, are attractive possibilities (figure 2). Ideally, these predictive models should stratify risk and inform which investigations and interventions should be considered. Uncertainties regarding who to target for predictive testing will need to be resolved. In high prevalence regions, can we justify offering testing to the whole population, with the attendant cost implications, or should testing be confined to high-risk groups, such as family members of IBD-affected individuals?

**Figure 2:**
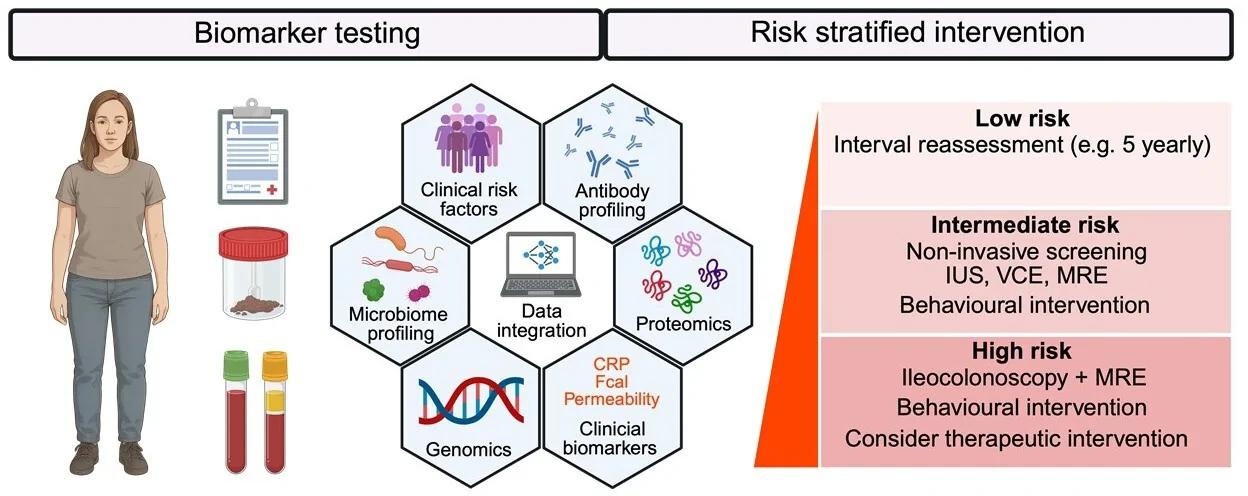
Development of biomarker panels for predicting IBD development and risk-based management frameworks. IBD, inflammatory bowel disease; IUS, intestinal ultrasound; MRE, magnetic resonance enterography; VCE, video capsule endoscopy.

## Management and intervention strategies

Following validation of reliable predictive assays, a framework for risk-based preventative interventions will need to be established (figure 2). Low risk individuals could be followed up for interval re-testing, whereas higher risk individuals would be directed towards screening or confirmatory diagnostic tests (figure 2). Discussing prophylaxis treatment options would also be appropriate in individuals at the highest risk of disease. In a screening setting, it may be more appropriate to offer non-invasive investigations that are likely to be more acceptable to patients, including intestinal ultrasound, MRI and video capsule endoscopy. Although there is a lack of evidence to inform which specific interventions might be implemented to prevent or delay onset of IBD, options include modifying behavioural risk factors and consideration of specific drug therapy. Proactive behavioural modification could be advised to most patients as this is likely a low-risk intervention and may even confer other collateral health benefits. Large-scale epidemiological studies and a few interventional studies have linked behavioural patterns, environmental exposures and dietary habits to altered risk of IBD or IBD flares.^31^ Therefore, advising avoidance of risk associated features (eg, smoking cessation and avoidance of ultra-processed or high sugar foods in patients at risk of CD) and advocating consumption of potentially protective features (eg, high fibre/Mediterranean diet) (figure 3) are relatively straightforward mitigation strategies and are likely to be acceptable to patients.

**Figure 3:**
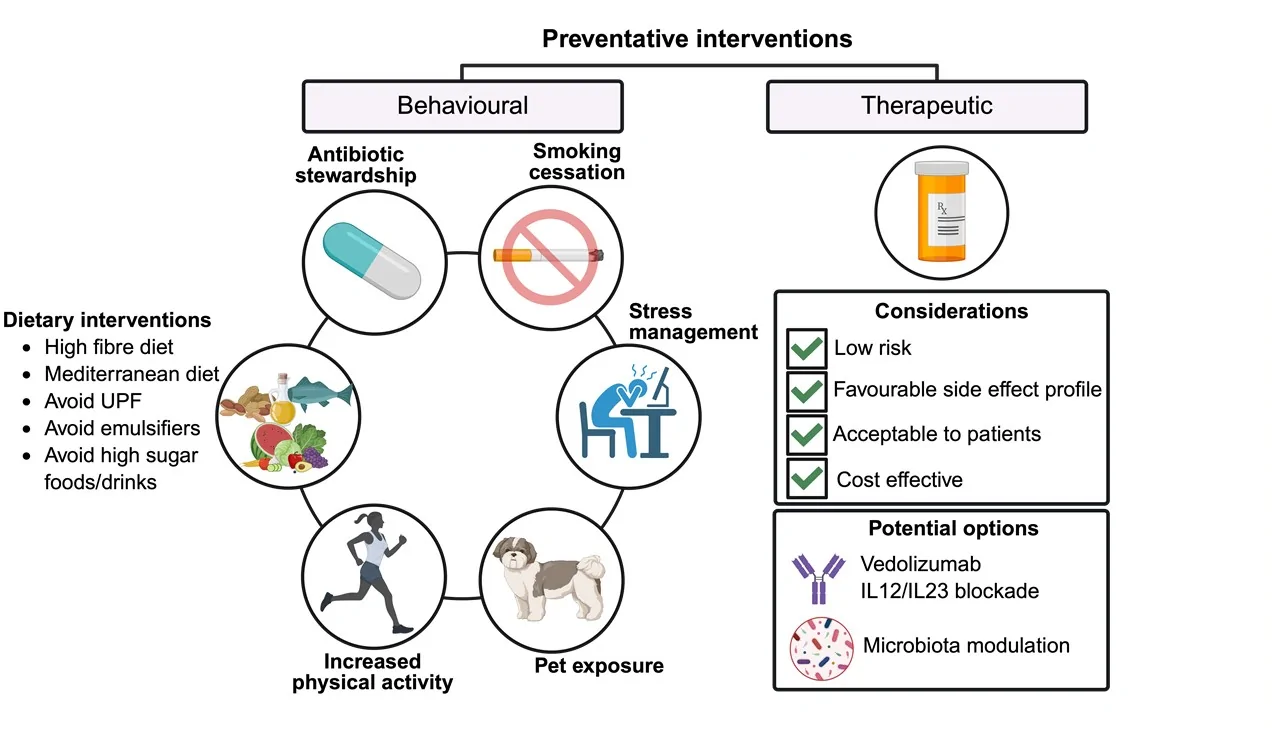
Preventative intervention strategies in preclinical IBD. IBD, inflammatory bowel disease.

In patients at the highest risk of IBD development, we need to establish whether interventions with specific drug therapy can prevent or delay IBD onset. There are encouraging precedents and valuable lessons emerging from analogous scenarios in other imuune-mediated inflammatory diseases (IMIDs). Just like in IBD, there are increased circulating autoantibodies, cytokines and cytokine receptors in the blood of patients with rheumatoid arthritis (RA), many years prior to diagnosis.^32 33^ The ground-breaking Apippra trial was a randomised, placebo-controlled phase 2a study that evaluated the efficacy, safety and acceptability of a preventative treatment strategy in the prediagnosis phase of a population of individuals at high risk of developing RA.^34^ Significantly fewer individuals progressed to develop RA following 12 months of treatment with abatercept in comparison with placebo-treated individuals.^34^ However, this effect was lost at 24 months, implying that ongoing long-term treatment may be needed to fully prevent the onset of RA. If therapeutic intervention is considered in pre-IBD, treatments that are low risk with minimal side effects and are acceptable to patients should be prioritised for clinical trials (figure 3). From currently available drugs, vedolizumab, ustekinumab or anti-IL23p19 monoclonal antibodies are attractive possibilities, but their efficacy in this setting is untested, especially if the immunologically active aspects of the initial triggering mechanisms are distinct from established IBD. Given the intestinal microbial changes observed prior to the development of CD, it is conceptually attractive to envisage that correcting predisease dysbiosis might reverse IBD susceptibility. Faecal microbiota transplantation is considered an acceptable treatment option for many patients and encapsulated oral formulations are especially favourably considered.^35^ Without being able to predict the subsequent severity and clinical course of future IBD, there is also a risk of overtreating patients who would have only developed mild IBD. Efforts to map preclinical biomarkers to subsequent disease trajectories and severity will be crucial to justify predisease treatment strategies. Some preclinical biomarkers, such as anti-GM-CSF autoantibodies, correlate with more severe disease and are associated with early progression to stricturing and penetrating phenotypes.^22^ Therefore, these features might be especially helpful in risk stratification. Should predisease therapies fail in clinical trials, or are deemed financially unviable by regulators, we now have evidence-based options of early aggressive treatment, at least in CD. The PROFILE study clearly demonstrated that very early treatment with infliximab and azathioprine, initiated within days of diagnosis, is a highly efficacious treatment strategy with high rates of mucosal healing in CD.^36^ With the implementation of highly effective advanced therapies at diagnosis, prolonged treatment of presymptomatic disease might no longer be necessary. However, long-term follow-up of IBD patients treated aggressively with optimised advanced therapies from diagnosis is needed to determine whether this approach genuinely alters the natural history of disease and prevents disease complications.

In summary, there is now sufficient optimism to believe that early detection of IBD, prior to the onset of symptoms, is an achievable and worthwhile goal. The IBD community is mobilising to tackle this important issue, and the EU funded INTERCEPT study (https://www.intercept-ihi.eu) seeks to verify and validate a biomarker panel to build a risk score for individuals with a high likelihood of developing CD. In the coming years, we will know just how well we can predict future IBD, and we will make our first attempts at formulating viable prevention strategies.
